# Relationship between Short-Range and Homotopic Long-Range Resting State Functional Connectivity in Temporal Lobes in Autism Spectrum Disorder

**DOI:** 10.3390/brainsci11111467

**Published:** 2021-11-05

**Authors:** Xiaoyin Wu, Fang Lin, Weiting Sun, Tingzhen Zhang, Huiwen Sun, Jun Li

**Affiliations:** 1 South China Academy of Advanced Optoelectronics, South China Normal University, Guangzhou 510006, China; xiaoyin.wu@coer-scnu.org (X.W.); fang.lin@coer-scnu.org (F.L.); weiting.sun@coer-scnu.org (W.S.); tingzhen.zhang@coer-scnu.org (T.Z.); huiwen.sun@coer-scnu.org (H.S.); 2 Key Lab for Behavioral Economic Science & Technology, South China Normal University, Guangzhou 510006, China

**Keywords:** autism spectrum disorder (ASD), functional near-infrared spectroscopy (fNIRS), resting-state functional connectivity (RSFC), short-range RSFC, long-range RSFC

## Abstract

To investigate the relationship between short-range and homotopic long-range resting state functional connectivity (RSFC) in children with autism spectrum disorder (ASD) and typically developing (TD) children, we analyzed functional near-infrared spectroscopy (fNIRS) RSFC in 25 children with ASD and 22 age-matched TD children. The resting state fNIRS signals, including spontaneous fluctuations in the oxygenated hemoglobin (HbO_2_) and deoxygenated hemoglobin (Hb) concentrations, were recorded from the bilateral temporal lobes. We found that (1) there was no difference in the short-range RSFC between the left and right hemisphere in either ASD or TD group; (2) both the short-range and homotopic long-range RSFC were weaker in the ASD than TD group; and (3) the short-range RSFC was stronger than the homotopic long-range RSFC in the ASD group, whereas no such difference was observed in the TD group. These observations might be helpful for a better understanding of the underlying cortical mechanism in ASD.

## 1. Introduction

Autism spectrum disorder (ASD) encompasses a wide range of neurodevelopmental disorders characterized by persistent deficits in social interaction, verbal and non-verbal communication, and non-social features such as repetitive or stereotypic behavior patterns and sensory abnormalities [1]. In March 2020, the US Federal Centers for Disease Control stated that 1 in every 54 children aged 8 years was affected by autism [2]. The rapid increase in the prevalence of ASD brings about a series of underlying challenges for ASD patients, their families and society, impacting their ability to take in standardized education, and to emotionally interact with their caregivers, families or peers. Evaluating and diagnosing ASD as early as possible is essential for assisting children with ASD in receiving clinical and societal services to reach their full potential capability [3]. The diagnosis of ASD is currently based on behavioral observations, e.g., via the Autism Diagnostic Observation Schedule (ADOS) [4,5], which relies on individuals who manifest consistent behaviors and habitual patterns, since inconsistent behaviors (such as those usually shown in younger children) may affect the diagnostic accuracy. For this reason, many neuroimaging researchers are dedicated to finding out characteristics associated with ASD in terms of brain structure and function that might serve as diagnostic physiological indicators for ASD.

Several recent imaging studies on ASD have demonstrated that social cognitive and communicative impairments are associated with alterations in the functional connectivity between several spatially segregated brain areas [6,7,8]. The functional connectivity reflects the degree of synchronization or correlation of the time series of brain signals originated from different cerebral regions [9,10]. In studying ASD with functional connectivity, a prevalent hypothesis proposed is that the autistic brain is characterized by over-connectivity in short-range areas and under-connectivity in long-range areas [11]. To assess this hypothesis, a number of imaging studies have been performed on ASD. Functional magnetic resonance imaging (fMRI) studies have revealed aberrant long-range resting-state functional connectivity (RSFC) in individuals with ASD, but with divergent findings in the direction of the alteration [10,12,13]. For instance, Dinstein et al. observed weaker long-range RSFC between the bilateral inferior frontal gyrus (IFG) and superior temporal gyrus (STG) in toddlers with autism than in the normal controls. STG is implicated in the processing of spoken word recognition, as well as in the visual analysis of social information conveyed by gaze and body movement, which is related to social functions such as theory of mind [14,15]. The reduced long-range RSFC between the bilateral STGs may be associated with ASD symptoms such as a lack of verbal communication and social interaction. Additionally, Anderson et al. also found reduced long-range connectivity between the inter-hemispheric homologous voxels of the superior parietal lobule, sensorimotor cortex, and STG in adults with ASD. However, Monk et al. reported stronger long-range connectivity between the posterior cingulate cortex and both the right temporal lobe and right parahippocampal gyrus, which may relate to the restricted and repetitive behaviors in ASD. In addition to fMRI, fNIRS, as an optical brain imaging modality, has been also utilized in the study of ASD. For example, two fNIRS studies have demonstrated that children with ASD show weaker long-range RSFC between the bilateral temporal lobes [16,17], consistent with Dinstein et al.’s finding. However, Kikuchi et al. observed long-range over-connectivity between the bilateral frontal lobes in children with ASD [18].

A few studies available to date have used functional magnetic resonance imaging (fMRI) and fNIRS to examine short-range connectivity, which is usually defined as the interaction between adjacent areas separated by a short distance (e.g., 6–30 mm; for reviews, see [7,17,19,20,21]) and within the same functional area. By contrast, the long-range connectivity is often defined as the connectivity between brain regions across different lobes, or as the temporal binding between local networks irrespective of location [6,22]. In short-range fMRI-RSFC analysis, Dajani and Uddin have observed weaker connectivity in children with ASD in the lateral occipital cortex and cerebellar lobule VI but stronger connectivity in the right precentral gyrus, STG, and IFG. Meanwhile, in another fMRI study, Long et al. demonstrated under-connectivity in the bilateral orbitofrontal cortex in children with ASD, and in the posterior cingulate cortex and medial prefrontal cortex in adolescents with ASD.

These findings on RSFC cannot consistently support the hypothesis that patients with ASD have long-range under-connectivity and short-range over-connectivity. Besides this, we noticed that most of the imaging studies evaluated long-range and/or short-range RSFC, and compared individuals with ASD and typically developing (TD) controls in terms of long- and/or short-range RSFC. Few studies, however, have directly investigated the relationship between short- and long-range RSFC in each of the individuals, including patients with ASD and their matched TD controls. Therefore, in this study, we investigated, with fNIRS, the relationship between short- and long-RSFC in each individual of children with ASD and TD children.

As an optical brain imaging modality, fNIRS has an excellent temporal and acceptable spatial resolution. Apart from this, fNIRS is more tolerant to motion artifacts caused by head movement than other brain imaging modalities such as fMRI and magnetoencephalography (MEG), which may render fNIRS a suitable alternative for studying RSFC in a more natural environment, since in recording spontaneous brain activity, the subjects have to keep their heads as motionless as possible for several minutes, e.g., 5–10 min, a typical time duration in an RSFC study. Thus, we utilized fNIRS to record the resting-state cerebral hemodynamic fluctuations, measure the short- and long-range RSFC, and then investigate their relationship for children including children with ASD and TD controls. Our hypothesis was that the relationship between the short- and long-range RSFC might be different between children with ASD and TD children. To test this hypothesis, we measured the short-range RSFC and homotopic long-range RSFC (i.e., the correlation between inter-hemispheric homologous regions) in the temporal lobes, quantitatively compared the two types of RSFC in children with ASD and TD children and each type of RSFC between the two groups, and finally evaluated the differences with analysis of variance (ANOVA).

## 2. Materials and Methods

### 2.1. Experimental Protocol and Participants

RSFC measurements were performed with a commercial continuous-wave fNIRS system (FOIRE-3000, Shimadzu Corporation, Kyoto, Japan) working at three wavelengths, 780 nm, 805 nm and 830 nm, with a sampling rate of 14.3 Hz. The absorptions of near-infrared light were then transformed into concentration changes for oxy-hemoglobin (HbO_2_), deoxy-hemoglobin (Hb) and total hemoglobin (HbT = HbO_2_ + Hb) according to the modified Beer–Lambert law. The source-detector distance was fixed at 30 mm.

**Figure 1:**
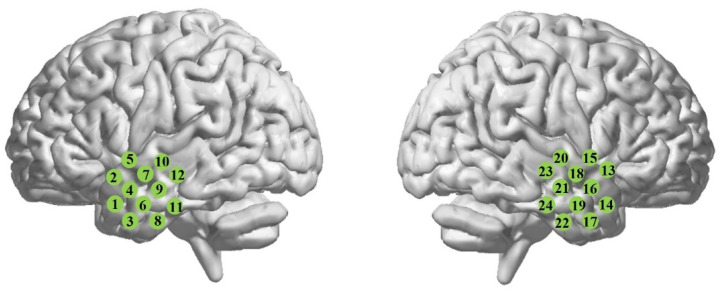
Schematic representation of channel locations on the bilateral temporal lobes.

During the experiment, the subjects were sitting in a comfortable chair in a dark and quiet room. They were asked to close their eyes, “do nothing” and try their best to minimize body and head movement. The spontaneous hemodynamic fluctuations were recorded form the bilateral temporal lobes (TLs) for approximately 8 min. Twenty-four optical channels were fixed on the scalp, with twelve channels at each hemisphere, as shown in Figure 1. To ensure good contact between the optode and scalp, we carefully treated hairs by using an ear pick with an LED light to push hairs aside until we clearly saw the skin (scalp), and then inserted the optode into the optode holder fixed in the FLASH (flexible adjustable surface holder). The scalp areas for the locations of the bilateral TLs were identified with reference to the international 10-10 system.

Twenty-five children with ASD (9.3 ± 1.4 years old; 18 boys and 7 girls) and twenty-two age-matched TD children (9.5 ± 1.6 years old; 18 boys and 4 girls) were recruited to participate in the experiment. They were all right-handed. All the children with ASD were diagnosed by experienced clinicians in hospitals based on DSM-IV-TR [1], while the TD children all had no history of any neurological disease. The IQ scores for all the participants were measured using the Raven’s Standard Progressive Matrices Test [23], resulting in IQ = 106 ± 12 for the TD and 91 ± 15 for the ASD groups. The difference in IQ between the two groups was significant (*p* < 0.05). It has been revealed that intelligence performance may be strongly associated with the functional connectivity between the frontal and other regions such as the parietal, occipital and limbic lobes [24,25,26,27,28]. No evidence has shown that IQ is associated with functional connectivity in the temporal lobes. Therefore, IQ could not be a factor for any difference in RSFC observed in this study.

Prior to the data acquisition, all the subjects were informed about the experimental procedure and written consent was obtained from the parents of the children. The study protocol was approved by the University’s Ethical Review Board at South China Normal University.

### 2.2. Data Analysis

Among the three measured hemodynamic variables, only HbO_2_ and Hb were analyzed, since HbT was the sum of HbO_2_ and Hb, not an independent variable. Even though the optodes were tightly fixed to the scalp and the subjects were asked to keep the head and body as motionless as possible, there still might have been some artifacts induced by head movement in the recorded fNIRS signals (i.e., the time series of HbO_2_ and Hb). To correct the motion artifacts, a wavelet algorithm used in the HOMER2 package, i.e., hmrMotionCorrectWavelet [29,30], was first applied to the raw time series of HbO_2_ and Hb. Secondly, the time series were detrended by using a second-order polynomial fit to remove slow drift, followed by using a band-pass (0.009–0.08 Hz) filter to get rid of most of the systemic hemodynamic components, such as those originated from cardiac cycles (~1 Hz), respirations (~0.2–0.3 Hz) and the Mayer waves (~0.1 Hz). To further correct motion artifacts and suppress low-frequency systemic interference, following the band-pass filter, we utilized an independent component analysis (ICA) algorithm [16,31]. This ICA-based approach can be shortly described as follows: we assume that the systemic component makes a similar contribution to each optical channel; one independent component is estimated as the systemic component as long as all the elements of its corresponding column in the mixing matrix A of ICA (Z = A × ICAs) have the same sign and least variation. A motion artifact in the signal usually manifests itself as a sudden drop (or rise) that can also be reflected in the independent components (ICs) showing similar temporal behavior. These ICs can be visually identified and then removed from the reconstruction of signal Z. In most cases, our experience showed that an IC could be identified as being associated with the head movement as long as the peak magnitude was over 10 times the standard deviation of the IC. These motion-related ICs can be eliminated from the matrix of ICAs; thus, the reconstructed signal Z no longer contains the artifacts.

**Figure 2:**
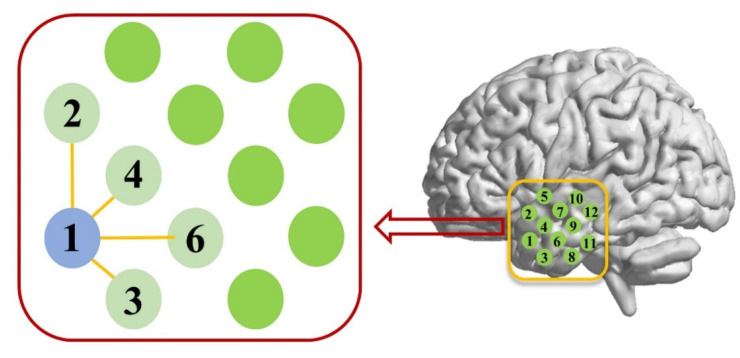
Schematic representation of the short-range RSFC. For example, there are 4 correlation coefficients for channel pairs formed by Channel-1 and its adjacent (distance ≤ 30 mm) channels including Channel-2, 3, 4, and 6. The average of the 4 correlation coefficients indicates the short-range RSFC for Channel-1. The distances between Channel 1 and Channel 2, and Channel 1 and Channel 6 were 30 mm. The distances between Channel 1 and Channel 3, and Channel 1 and Channel 4 were 21 mm.

After those above-mentioned data-preprocessing steps, an N-by-N correlation matrix (with N being the number of optical channels) was computed, in which each element was the Pearson correlation coefficient for each optical channel pair, reflecting the RSFC between the two corresponding channels of the pair. In the present study, the RSFC was classified into two categories: the short-range and homotopic long-range RSFC. The short-range RSFC was defined as the RSFC locating at the same temporal lobe and with a distance between 21 and 30 mm, as shown in Figure 2. For each channel (e.g., Channel-k, k = 1:24), there were 4 or 7 short-range connections between Channel-k and its neighboring (with a distance ≤ 30 mm) channels, yielding 4 or 7 correlation coefficients. The average of these correlation coefficients indicated the short-range RSFC for Channel-k. Therefore, for the left (or right) hemisphere, there were 12 short-range RSFCs, which could be averaged to obtain the average of the short-range RSFC for the left (or right) hemisphere. The long-range RSFC was defined as the mirrored homotopic RSFC between the bilateral temporal lobes. For the long-range RSFC, there were 12 inter-hemispheric connections, corresponding to 12 correlation coefficients. Therefore, the average of the long-range RSFC was the average over the 12 correlation coefficients. To compute the average of the correlation coefficients, Fisher’s r-z transform was used, in which each correlation coefficient r was first converted to its z-score, and then, the average across all the z-scores was calculated.

A two-way ANOVA was applied to the RSFC data including the average of the short-range for the left and right hemispheres with diagnosis (two levels: ASD and TD) as the between-subject factor, and hemisphere as the within-subject factor to investigate the short-range RSFC. To investigate whether the difference in functional connectivity between the short range and homotopic long range was significant, another two-way ANOVA was employed with diagnosis (two levels: ASD and TD) as the between-subject factor, and different types of RSFC (two levels: the short range and long range) as the within-subject factor. In this study, we defined significance by two criteria: *p* < 0.05 and statistical power (1-*β*) > 0.8, which could effectively restrain the type Ⅰ (*α*) and type Ⅱ errors (*β*).

## 3. Results

### 3.1. Short-Range RSFC at the Temporal Lobes

**Figure 3:**
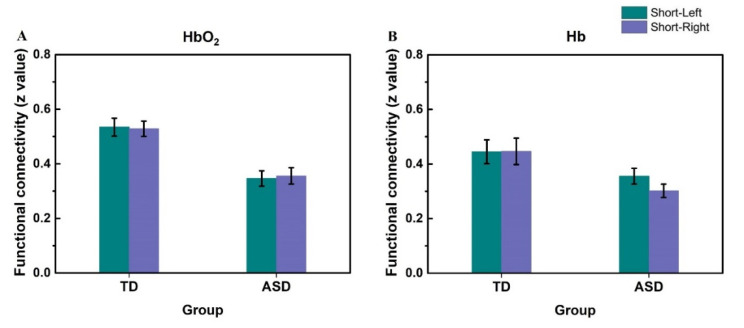
The average of short-range RSFC at two hemispheres for HbO_2_ (**A**) and Hb (**B**) for ASD and TD groups. Short-Left: the short-RSFC on the left hemisphere. Short-Right: the short-RSFC on the right hemisphere. The error bar is the standard error of the mean. For HbO_2_-RSFC, in the TD group, the z-value was 0.53 ± 0.03 for Short-Left, and 0.53 ± 0.03 for Short-Right; while in the ASD group, the z-value was 0.35 ± 0.03 for Short-Left, and 0.36 ± 0.03 for Short-Right. For Hb-RSFC, in the TD group, the z-value was 0.44 ± 0.04 for Short-Left, and 0.45 ± 0.05 for Short-Right, while in the ASD group, the z-value was 0.36 ± 0.03 for Short-Left, and 0.30 ± 0.02 for Short-Right. In each group, there was no significant difference between the left and right hemispheres in short-RSFC for either HbO_2_ or Hb.

Two-way ANOVA with the factors of diagnosis (ASD or TD) and the hemisphere region (the left or right temporal lobe) was performed on the short-range RSFC in HbO_2_. The results are shown in Table 1, indicating that, for the short-range RSFC, neither the main effect of the hemisphere nor the interaction between the hemisphere and diagnosis was significant. However, the main effect of diagnosis on the short-range RSFC was statistically significant, indicating there was a significant difference in short-range RSFC between the ASD and TD groups. Figure 3A shows the short-range HbO_2_-RSFC for the left and right hemisphere for the two groups. Since there was no difference in the short-range RSFC between the left and right hemisphere, we could calculate the mean across the two hemispheres as the averaged short-range RSFC for each individual. 

The two-way ANOVA on the short-range Hb-RSFC led to a similar result to that for HbO_2_-RSFC. Figure 3B shows the average of the short-range Hb-RSFC at each hemisphere. No significant difference between the left and right temporal lobes was found (partial η^2^ = 0.029, *F*_(1, 45)_ = 1.344, *p* = 0.252, and 1-*β* = 0.206). Therefore, for the short-range Hb-RSFC, we could also calculate the mean across the two hemispheres as the averaged short-range RSFC for each individual.

### 3.2. The Difference between the Short-Range and Homotopic Long-Range RSFC

**Figure 4:**
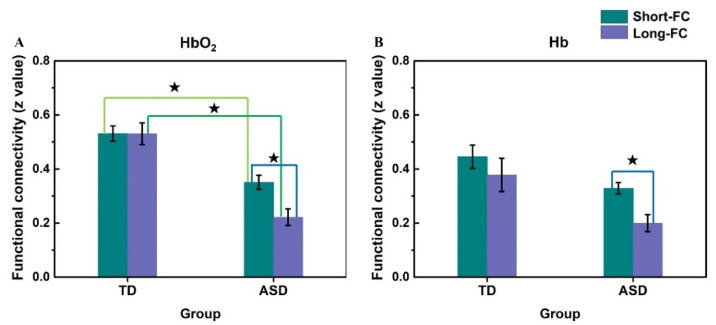
The average of short-range RSFC (Short-FC) and homotopic long-range RSFC (Long-FC) based on HbO_2_ (**A**) and Hb (**B**) in the bilateral temporal lobes for ASD and TD groups. The error bar is the standard error of the mean. For HbO_2_-RSFC, in the TD group, the z-value was 0.53 ± 0.03 for Short-FC, and 0.53 ± 0.04 for Long-FC, while in the ASD group, the z-value was 0.35 ± 0.03 for Short-FC, and 0.22 ± 0.03 for Long-FC. For Hb-RSFC, in the TD group, the z-value was 0.45 ± 0.04 for Short-FC, and 0.38 ± 0.06 for Long-FC, while in the ASD group, the z-value was 0.33 ± 0.02 for Short-FC, and 0.20 ± 0.03 for Long-FC. The short-RSFC was significantly stronger than the long-RSFC in the ASD group in both HbO_2_ and Hb, whereas, in the TD group, there was no significant difference between the short- and long-RSFC in either HbO_2_ or Hb. For HbO_2_, both the short- and long-RSFC were weaker in the ASD than TD group. The symbol ★ indicates the difference is significant.

The HbO_2_-RSFC (short- and long-range) were subjected to a two-way ANOVA with the factors of diagnosis (ASD or TD) and the type of RSFC (the short- or long-range). Table 2 shows the ANOVA results, indicating there was a significant interaction between the RSFC type and diagnosis. Simple effect analysis showed that there was a statistically significant difference between the short-range and long-range RSFC in the ASD group (partial η^2^ = 0.406, *F*_(1, 45)_ = 30.780, *p* < 0.001, and 1-*β* = 0.999), but no such difference in the TD group (partial η^2^ = 0.000031, *F*_(1, 45)_ = 0.001, *p* = 0.970, and 1-*β* = 0.050), as shown in Figure 4A. In addition, the ASD group had significantly weaker short-range (partial η^2^ = 0.329, *F*_(1, 45)_ = 22.074, *p* < 0.001, and 1-*β* = 0.996) and homotopic long-range (partial η^2^ = 0.461, *F*_(1, 45)_ = 38.488, *p* < 0.001, and 1-*β* = 0.999) RSFC than the TD group.

A two-way ANOVA was also conducted on Hb-RSFC with two factors including the diagnosis (ASD or TD) and RSFC type (the short-range or long-range type). Table 3 shows the ANOVA results indicating there were significant main effects for both diagnosis and RSFC type, but no interaction effect between the two factors. Further analysis revealed there was a significant difference in Hb-RSFC between the short- and long-range types in the ASD group (partial η^2^ = 0.284, *F*_(1, 45)_ = 17.813, *p* < 0.001, and 1-*β* = 0.985), but no significant difference in the TD group (partial η^2^ = 0.087, *F*_(1, 45)_ = 4.261, *p* = 0.045, and 1-*β* = 0.524), as illustrated in Figure 4B. Although the TD group showed a difference with a small p-value, i.e., *p* = 0.045 < 0.05, the statistical power (1-*β*) = 0.524 was not big enough (e.g., < 0.8) to demonstrate a significant difference with the current sample size. The weaker Hb-RSFC in both the short-range and homotopic long-range types were observed in the ASD group as compared to the TD group; however, the statistical powers for the differences in Hb-RSFC between the two groups were slightly less than 0.8 (partial η^2^ = 0.124, *F*_(1, 45)_ = 6.359, *p* = 0.015, and 1-*β* = 0.694 for short-range; partial η^2^ = 0.138, *F*_(1, 45)_ = 7.216, *p* = 0.010, and 1-*β* = 0.748 for homotopic long-range); therefore, these differences were not considered here as significant alterations.

## 4. Discussion

In this study, the source–detector distance was fixed at 30 mm. Considering that the total thickness of the scalp and skull in the measured temporal areas for the children was around 9 mm [32,33], it was possible for the near-infrared light to probe the cortex. However, systemic interference such as that originated from the scalp was inevitably contained in the recorded fNIRS signals, which could not be eliminated by simply applying a band-pass filter, thus inducing variation in the calculation of the correlation coefficients. A more effective way is to use a separate channel with a short source–detector (SD) spacing (e.g., ~10 mm) to only detect the interference from the scalp, which can then be regressed out from the fNIRS signals recorded with long SD spacing (e.g., 30 mm). In this way, the signal from the cortex can be reliably detected. However, our commercial fNIRS setup was not equipped for short SD detection. To obtain the cortical activity from the fNIRS signal mixed with the interference, we utilized an ICA-based algorithm that could suppress the systemic interference. By this approach, we obtained the temporal RSFC in TD children and children with ASD, and observed significant alterations in RSFC between the two groups. Though the systemic interference might not have been completely excluded by the algorithm, it could not be a reason for the difference in the measured RSFC between the TD and ASD groups, since, to date, there is no study reporting differences in the scalp hemodynamics (e.g., blood flow or blood oxygenation level), but there are many studies demonstrating RSFC changes in the brain between the two groups.

The temporal lobes, which are implicated in autism, showed significantly lower strength in both the short- and homotopic long-range RSFC in the children with ASD than in the TD children. Moreover, our results revealed that children with ASD showed a significant difference between the short- and homotopic long-RSFC for both HbO_2_ and Hb, whereas in the TD children, there was no such difference, indicating that the TD children showed similar strength in the short- and homotopic long-range RSFC (see Figure 4). These results may provide evidence for supporting our proposed hypothesis that the relationship between the short- and long-range RSFC might be different between children with ASD and TD children.

In previous autism studies with fMRI [7] and fNIRS [17], reduced short-range RSFC has been observed in children with ASD, in particular, in the orbitofrontal cortices and temporal cortices, in line with our observation in the present study. However, in another fMRI study, Dajani and Uddin [21] observed short-range over-connectivity in the right precentral gyrus, STG, and IFG. To find a possible reason for the discrepancy, we compared the distance range for short-range RSFC in these studies and found that the distance used for defining the short-range RSFC was not consistent, spanning a rather wide range from 6 to 30 mm. When the distance was in the range of 10–30 mm (e.g., 10–30 mm in Long et al.’s study; 21–30 mm in Zhu et al.’s and our studies), under-connectivity was observed in children with ASD. However, when the distance was short, e.g., 6 mm in Dajani and Uddin’s study [21], over-connectivity was observed in brain regions such as STG and IFG, both locating in the temporal lobe. Therefore, it seems that the alteration (over- or under-connected) in short-range connectivity relies on the distance used for defining the “short range”, which is, unfortunately, not well-defined and may have different values across different studies. This might be a reason for inconsistent observations for the alteration of short-range RSFC in ASD.

The weaker homotopic long-range RSFC in the bilateral temporal lobes has been previously observed in ASD [10,13,34]. In addition, it has been reported that the ASD symptom severity as measured using ADOS is negatively associated with the homotopic long-range RSFC in the posterior cingulate (r = −0.189) and STG (r = −0.220), implying the more the reduction in the homotopic long-range RSFC, the more the ASD symptom severity [34]. Overall, the present study does support long-range under-connectivity but does not support short-range over-connectivity in ASD, which is basically in line with the conclusion obtained by analyzing the whole-brain intrinsic functional connectivity that ASD-related hypoconnectivity is more dominant and robust [35].

The short- and long-range RSFC have usually been studied by a comparison between ASD and TD groups to find ASD-related alterations in the short- and long-range RSFC. Few studies have examined the relationship or difference between the short- and long-range RSFC in each individual. In the present study, we used fNIRS to investigate the relationship between the short- and homotopic long-range RSFC in children with ASD and TD children. We found under-connectivity in children with ASD in both short- and long-range RSFC as compared to the TD controls. Besides that, the children with ASD exhibited significantly stronger short- than long-range RSFC (see Figure 4), whereas in the TD children, there was no such difference between the short- and long-RSFC. This observation might imply that the functional networks in the temporal lobes in ASD are impaired over a wide-length scale, e.g., from a short distance (e.g., 21 mm) for local connection to several tens of centimeters for inter-hemispherical connection, and the longer the distance, the weaker the connectivity (e.g., more loss of the coherence of the spontaneous activity in functionally related cortical sites). This characteristic may be useful for a better understanding of the underlying hemodynamic mechanism in ASD.

fNIRS, as a non-invasive brain mapping technique, has some unique advantages such as relatively low sensitivity to head movement and no special requirements for the experimental environment (i.e., it can be performed in a more natural environment), thus providing a suitable imaging tool with which to study the brains of humans, particularly young children. It has been demonstrated that the homotopic RSFC can be used for differentiation between children with ASD and TD children [16]. The combination of more characteristics associated with ASD may be beneficial for a more accurate prediction of ASD. In the present study, we defined significant differences by using two criteria: *p* < 0.05 and statistical power (1-*β*) > 0.8. This was probably effective for restraining type I and type II errors of statistical analysis with the current sample size (25 children with ASD and 22 TD children), ensuring that the results obtained were more reliable and robust.

## 5. Conclusions

In addition to the weaker homotopic long-range RSFC between the bilateral temporal lobes, which has been well established in ASD studies, in the present study, we also observed weaker short-range RSFC in the temporal lobes in ASD, not in line with the prevalent hypothesis in which short-range over-connectivity is assumed, but in line with Di Martino et al.’s finding that ASD-related hypoconnectivity is more dominant in the whole-brain intrinsic functional connectivity [35]. Further study on the relationship between the short- and homotopic long-range RSFC has revealed that there is no difference between the short- and long-range RSFC in TD children, whereas children with ASD show stronger short- than long-range RSFC. This new characteristic may be helpful for a better understanding of the underlying cortical mechanism of the autistic brain, and useful for a more accurate prediction of ASD.

## Figures and Tables

**Table 1:** Main effect of diagnosis, main effect of hemisphere, and hemisphere–diagnosis interaction revealed by two-way ANOVA for short-range HbO_2_-RSFC.

Factor	df	Partial η^2^	*F*-Value	*p*-Value	(1-*β*) Value
diagnosis	(1, 45)	0.329	22.074	<0.001	0.996
hemisphere region	(1, 45)	0.0002	0.010	0.923	0.051
hemisphere region–diagnosis interaction	(1, 45)	0.004	0.199	0.658	0.072

**Table 2:** Main effect of diagnosis, main effect of RSFC type, and type–diagnosis interaction revealed by two-way ANOVA for HbO_2_-RSFC.

Factor	df	Partial η^2^	*F*-Value	*P*-Value	(1-*β*) Value
diagnosis	(1, 45)	0.441	35.467	<0.001	0.999
RSFC type	(1, 45)	0.245	14.616	<0.001	0.962
RSFC type–diagnosis interaction	(1, 45)	0.240	14.201	<0.001	0.958

**Table 3:** Main effect of diagnosis, main effect of RSFC type, and type–diagnosis interaction revealed by two-way ANOVA for Hb-RSFC.

Factor	df	Partial η^2^	*F*-Value	*p*-Value	(1-*β*) Value
diagnosis	(1, 45)	0.148	7.837	0.008	0.782
functional connectivity type	(1, 45)	0.300	19.299	<0.001	0.990
functional connectivity type-by-diagnosis interaction	(1, 45)	0.041	1.910	0.174	0.272

## Data Availability

The data in the present study will be available upon reasonable request from the corresponding author and must be subject to the privacy restrictions and Regulations on Human Genetic Resources Management of China.
